# Impacts of *Andrographis paniculata* supplementation on health and productivity in monogastric farm animals: A comprehensive review

**DOI:** 10.1016/j.aninu.2025.07.004

**Published:** 2025-10-06

**Authors:** Elżbieta Redlarska, Małgorzata Ożgo, Mariusz Pierzchała, Katarzyna Kępka-Borkowska, Katarzyna Chałaśkiewicz, Chandra Shekhar Pareek, Adam Lepczyński

**Affiliations:** a Department of Physiology, Cytobiology and Proteomics, West Pomeranian University of Technology, Klemensa Janickiego 29 Str., Szczecin 71-270, Poland; b Department of Genomics and Biodiversity, Institute of Genetics and Animal Biotechnology of the Polish Academy of Sciences, Postępu 36A Str, Jastrzębiec, Magdalenka 05-552, Poland; c Department of Basic and Preclinical Sciences, Institute of Veterinary Medicine, Faculty of Biological and Veterinary Sciences, Nicolaus Copernicus University, Toruń 87-100, Poland

**Keywords:** Plant extract, Pig, Poultry, Green chiretta, Creat, Feed additive

## Abstract

Growing concerns about antibiotic resistance and the pursuit for natural alternatives in animal production have driven the search for dietary additives that promote health and sustainability, without adversely affecting the environment or consumer safety. One such alternative could be *Andrographis paniculata* (AP), a medicinal plant known for its bioactive compounds with anti-inflammatory, antioxidant, antimicrobial, antiviral and hepatoprotective properties. This review explores the potential of AP supplementation in improving the health and productivity of monogastric livestock, particularly poultry and swine. Key areas of focus include its beneficial effects on the immune and digestive systems, liver function and metabolism, factors that collectively support improved growth performance and production traits. Notably, the review highlights the efficacy of AP supplementation in mitigating the adverse impacts of environmental and physiological stressors such as heat stress, mycotoxin contamination, and microbial infections. Although the current findings underscore the promising potential of AP as a sustainable feed additive, further investigations, particularly in swine, are crucial to fully elucidate its mechanisms of action and broaden its practical applications in animal production systems.

## 1 Introduction

The use of antibiotics in animal feed, particularly at sub-therapeutic doses, has long been a common practice to promote growth and prevent disease in livestock. However, this widespread use has led to the emergence of antibiotic-resistant bacteria, a major global health issue. In addition, the misuse of antibiotics in agriculture and animal production can contribute to the accumulation of antibiotic residues in meat, milk and other animal products, posing health risks to consumers (Haque et al., 2023). These concerns have led to strict regulations in many countries, with the European Union banning antibiotics as growth promoters in 2006 (regulation 1831/2003/EC on additives for use in animal nutrition). Despite these efforts, antibiotic use in livestock farming remains high, particularly in regions where regulations are less strict or poorly enforced.

Over the past decade, the application of plant-based additives in the prevention and recovery of animal health has grown significantly due to increasing concerns over drug-resistant pathogens, the high costs associated with synthetic inputs and risk of toxic residues in feed products (Kuralkar and Kuralkar, 2021). As a result, natural alternatives such as herbal extracts are increasingly being employed to enhance animal health and food safety, providing a more sustainable and eco-friendly approach to livestock farming. One such herb is *Andrographis paniculata* (AP), a plant rich in bioactive compounds and widely used in traditional medicine in Asia.

The article focuses on monogastric animals, such as poultry and pigs, whose digestive systems differ significantly from those of ruminants. Unlike ruminants, which have complex multi-chambered stomachs, monogastric animals possess a single-chambered stomach and rely more on enzymatic digestion. This makes them particularly sensitive to the types of feed additives used, such as antibiotics or natural plant extracts. Additionally, the metabolism and gut microbiota of monogastric animals respond differently to these substances, affecting their health, growth, and disease resistance in ways that are distinct from ruminants. Therefore, focusing on monogastric animals allows for a more targeted discussion, addressing specific challenges and opportunities within this group. This review provides a comprehensive synthesis of current knowledge regarding the use of AP in monogastric livestock, with a focus on poultry and swine. It is structured around three main objectives: 1) to summarize the phytochemical profile and biological mechanisms of action of AP and its bioactive compounds, including anti-inflammatory, antiviral, anticancer and hepatoprotective pathways; 2) to review and discuss the effects of AP supplementation on health and productivity in poultry and swine across different production stages; and 3) to evaluate the implications of formulation, dose, and delivery methods, highlighting limitations in current research and identifying areas for further investigation.

## 2 Overview of AP

### 2.1 Origin and geographic distribution

AP belongs to the Acanthaceae family and is commonly known as “king of bitters” or “Kalmegh” in India (Ghosh et al., 2012). It can be found in tropical and subtropical regions of Asia, including Southeast Asia and some other countries like Cambodia, the Caribbean islands, Indonesia, Laos, Malaysia, Myanmar, Sri Lanka, Thailand and Vietnam (Hossain et al., 2014). This plant grows in warm, humid climates, usually in shady lowland areas. Historically, AP was cultivated on a small scale for domestic use, but its cultivation has expanded significantly, especially in India and China due to its important role in the pharmaceutical industry (Jiang et al., 2021).

### 2.2 Plant description

**Fig. 1:**
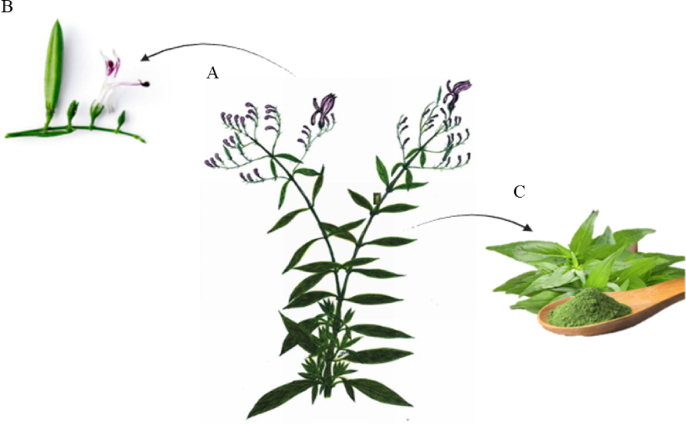
Morphology of *Andrographis paniculata*. (A) Whole plant; (B) flower; (C) leaves.

*A. paniculata* (Fig. 1A) is an annual herb that reaches from 30 to 110 cm height with a slender, slightly branched stem and small, lance-shaped leaves that range from 2 to 10 cm in length (Mishra et al., 2007; Hossain et al., 2014). The plant produces small white or light purple flowers arranged in panicle-like clusters (Fig. 1B) that bloom in narrow capsule-shaped fruits containing numerous tiny seeds (Jarukamjorn and Nemoto, 2008). The parts used medicinally include the leaves (Fig. 1C) and the entire herb, from which extracts and powders are derived (Kumar et al., 2021).

### 2.3 Traditional uses

In various traditional medical systems across Asia, AP has been highly valued for its broad therapeutic potential. It has been used primarily as an immune-boosting remedy and for treating a wide range of infections, including respiratory tract infections (Coon and Ernst, 2004; Hu et al., 2018; Poolsup et al., 2004), different types of fevers (Amaryan et al., 2003; Gupta et al., 2021) and digestive disorders (Choudhury et al., 2015; Wang et al., 2021). In traditional Chinese medicine, preparations from this herb are used to cleanse the blood and to “clear heat” from the body—meaning it is used as an antipyretic and anti-inflammatory agent (Hu et al., 2018). In Ayurvedic medicine, AP is also utilized to treat skin diseases (Sule et al., 2010), malaria (Mishra et al., 2009; Widyawaruyanti et al., 2014) and snake bites (Nayak et al., 2020; Premendran et al., 2011). The therapeutic effects are due to the bioactive compounds present in AP, but phytochemicals widely differ in terms of the part used, geography, season and time of harvesting (Hossain et al., 2014).

## 3 Bioactive compounds of AP and their therapeutic mechanisms

*A. paniculata* contains a variety of bioactive compounds, primarily diterpenoids, flavonoids, and polyphenols (Koteswara Rao et al., 2004). Among these, andrographolide (AG) is the most extensively studied and is responsible for many of the plant's pharmacological effects.

### 3.1 Andrographolide

Andrographolide is the main diterpenoid lactone isolated from AP and is responsible for its bitter taste (Boopathi, 2000; Zhang et al., 2018). This compound has been associated with a wide range of medicinal properties, including anti-inflammatory, antiviral, anticancer, antioxidant and hepatoprotective activities (Wang et al., 2021).

#### 3.1.1 Anti-inflammatory activity

Andrographolide demonstrates its anti-inflammatory effects by the regulation of key molecular signaling pathways such as nuclear factor kappa-light-chain-enhancer of activated B (NF-κB) cells (Hidalgo et al., 2005) and Janus kinase-signal transducer and activator of transcription (JAK-STAT) (Ding et al., 2017). Suppression of these pathways by AG decreases the synthesis of pro-inflammatory cytokines such as tumor necrosis factor-α (TNF-α), interleukin-6 (IL-6), interleukin-1 β (IL-1β) and enzymes like cyclooxygenase-2 (COX-2) (Tan et al., 2017), which results in a reduction of inflammatory response. This dual effect helps in mitigating oxidative damage in cells, protecting tissues from inflammation-induced injury. Moreover, AG's inhibition of COX-2 is significant, as this enzyme is integral to the production of prostaglandins, lipid compounds that mediate inflammation and fever (Jiao et al., 2019). By reducing COX-2 levels, AG not only alleviates inflammation but also decreases associated symptoms, such as pain and edema. AG also affects macrophage polarization, shifting these immune cells from a pro-inflammatory (M1) phenotype towards an anti-inflammatory (M2) phenotype, which is associated with tissue repair and regeneration (Li et al., 2022). By altering macrophage activity, AG promotes an environment that favors healing and limits chronic inflammation.

#### 3.1.2 Antiviral activity

In addition to the general anti-inflammatory effect, AG has shown the ability to inhibit the replication of various viruses, including influenza, hepatitis C, dengue, and even HIV and SARS-CoV-2 (Adiguna et al., 2021). AG's antiviral capabilities are rooted not only in its ability to modulate immune responses but also in its direct action on viral mechanisms. This bioactive compound can enhance the production of antiviral interferons by cytotoxic T-cells, natural killer cells and induce phagocytosis (Gupta et al., 2017). Additionally, AG's direct inhibition of viral protein synthesis disrupts the viral replication cycle at multiple stages (Hariprasath et al., 2022; Tan et al., 2022). By interfering with the synthesis of key structural and non-structural proteins, AG prevents viruses from successfully assembling and maturing within host cells, leading to incomplete or non-infective viral particles. AG's interference with virus-host cell entry adds another critical layer to its antiviral effects. By blocking receptor–ligand interactions, AG prevents viruses from attaching to or entering host cells, effectively stopping the infection at its point of entry (Jantan et al., 2023; Lim et al., 2021).

#### 3.1.3 Anticancer activity

Beyond its antiviral activity, AG's multifunctional properties extend into the realm of cancer prevention and treatment. It is suggested that AG may have anticancer properties due to its different mechanisms of inhibiting cancer cell proliferation. The same pathways that AG modulates to combat viral infections, such as NF-κB, JAK-STAT, and the enhancement of immune cell activity, are also central to its anticancer effects.

It has also been reported that AG has the ability to promote apoptosis by regulating pro-apoptotic proteins like Bcl-2-associated X protein (BAX) (Yang et al., 2010) or downregulating anti-apoptotic proteins, such as the B-cell lymphoma 2 (Bcl-2) family (Zhou et al., 2006), which are often overexpressed in cancer cells to evade programmed cell death. Beyond apoptosis, AG has the ability to inhibit angiogenesis by downregulation of vascular endothelial growth factor (VEGF) expression (Malik et al., 2021).

#### 3.1.4 Hepatoprotective activity

Andrographolide also exhibits potent hepatoprotective properties, safeguarding liver cells from damage due to toxins, oxidative stress, and inflammation. The same mechanisms that allow AG to inhibit cancer cell growth—such as its antioxidant, anti-inflammatory, and pro-apoptotic effects—also protect liver cells from injury caused by various stressors and pathological conditions, such as drug-induced hepatotoxicity, viral hepatitis, and fatty liver disease.

The hepatoprotective effect is achieved primarily by activation of mechanisms related to the prevention and reduction of oxidative stress (Bardi et al., 2014). It is manifested mainly by enhancement of the activity of superoxide dismutase and catalase, while reducing lipid peroxidation in liver tissues (Chua, 2014). This enhanced reactive oxygen species detoxification capacity not only protects against chemical and drug-induced liver injury but also promotes liver regeneration after damage.

### 3.2 Neoandrographolide

Neoandrographolide (NAG) is another diterpenoid that shares structural similarities with AG but has distinct biological activities. NAG exhibits strong free radical scavenging abilities, which help neutralize reactive oxygen species (ROS) (Liu et al., 2007). By inhibiting lipopolysaccharide (LPS)-induced nitric oxide (NO) production in mouse macrophages (Gong et al., 2018), NAG reduces the formation of reactive nitrogen species (RNS). This reduction in NO and RNS production helps mitigate inflammatory responses in tissues and protects cells from inflammation-induced oxidative stress. Furthermore, NAG may downregulate the expression of toll-like receptor 4 (TLR4) and NF-κB, the elements of the pro-inflammatory cascade, reducing systemic inflammation and immune overactivation (Gong et al., 2018). These combined effects highlight NAG's potential as a multi-targeted agent for managing inflammatory diseases, oxidative stress-related conditions, and immune-mediated pathologies, offering a promising alternative or complement to AG in therapeutic applications.

### 3.3 Andrograpanin

Andrograpanin (AGP) is a derivative of AG and shows similar, although less pronounced, biological properties. AGP is effective in inhibiting the production of NO, pro-inflammatory cytokines (Liu et al., 2008), and in the modulation of the NF-κB pathway (Yin et al., 2022). Thus, it helps to control inflammatory responses and limit tissue damage due to excessive immune activation. It is also reported that AGP exhibits significant inhibition of microbial biofilm production (Majumdar et al., 2020). By inhibiting biofilm formation, AGP disrupts this protective layer, making bacteria more susceptible to immune clearance and antibiotic treatment.

### 3.4 Other compounds

**Fig. 2:**
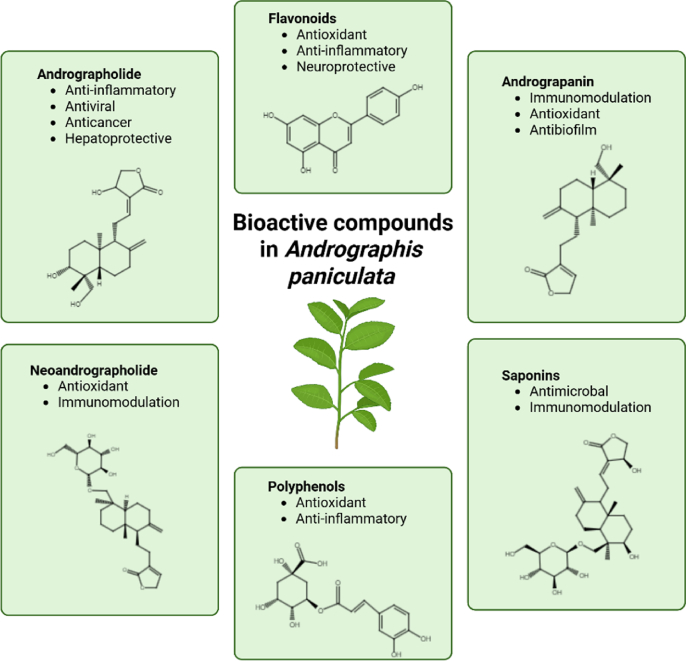
Main bioactive compounds found in *Andrographis paniculata*.

The diverse array of bioactive compounds in AP creates a synergistic effect that enhances its therapeutic potential, allowing it to address multiple health conditions through different mechanisms. Flavonoids found in AP, such as apigenin, luteolin and rutin (Annapareddy, 2012), have widely recognized antioxidant properties (Bodewes et al., 2011). These bioactive compounds also exhibit neuroprotective effects (Ishola et al., 2021). Also, polyphenols such as hydroxybenzoic and hydroxycinnamic acids, present in AP in smaller amounts, contribute to the overall antioxidant and anti-inflammatory activities of this medicinal plant (Paraveen, 2017). Saponins that are also found in AP have surfactant properties that can disrupt microbial cell membranes (Kee et al., 2023) contributing to antimicrobial activity. The diverse bioactive compounds found in AP exhibit a broad spectrum of medicinal properties, that could be used to promote overall health and prevent chronic diseases. The summary of bioactive compounds found in AP and their effects are shown in Fig. 2.

## 4 Effects of AP supplementation in poultry

As concerns rise over antibiotic resistance, consumer demand for organic and natural products has led to significant interest in phytogenic (plant-based) feed additives as safe and effective alternatives. The use of herbal extracts in poultry nutrition is an emerging approach aimed at enhancing growth, immunity and overall health. Among many herbs, AP is widely studied in poultry, especially in chickens and ducks. Supplementing poultry diets with AP has emerged as a promising strategy to improve growth performance, immunity and disease resistance.

### 4.1 Impact on the digestive system

Proper gut structure and function, which include digestion, nutrient absorption, barrier function, and microbiota composition, are critical to achieve optimal animal growth and productivity. AP supplementation positively influences the gastrointestinal tract morphology, digestive enzyme activity and intestinal microbiota composition. It was found that ingestion of AP impacts the morphology of the intestinal lining, specifically villus height, villus width, villus surface area, and crypt depth in ducks (Liu et al., 2023) and in progeny of an endemic chicken breed (Sujatha et al., 2015). Higher and wider villi increase the surface area of nutrient absorption, allowing more efficient uptake of essential nutrients, vitamins and minerals (Wang et al., 2020), indicating that AP can improve nutrient absorption. Moreover, proper gut morphology reduces the permeability of the intestinal barrier, decreasing the risk of pathogen translocation from the gut to host tissues.

The antimicrobial properties of AP make it a powerful modulator of gut microbiota composition. AP balances gut microbiota by selective inhibition of pathogenic bacteria such as *Escherichia coli* and *Salmonella* spp. and promotion of beneficial lactic acid bacteria in the small intestine (Hasanain et al., 2023; Jahja et al., 2023) and cecum (Liu et al., 2023). This balance in the gut microbiota is critical for prevention of dysbiosis which can further lead to digestive issues and decreased nutrient absorption. A balanced microbiota supports various digestive functions, including the breakdown of complex dietary components that cannot be digested by the endogenous enzymes of poultry gastrointestinal tract (Pan and Yu, 2014). Beneficial bacteria also produce short-chain fatty acids (SCFAs) like butyrate, which nourish the gut lining, reduce inflammation, and provide an additional energy source for the host (Segura-Wang et al., 2021). AP supplementation by promoting the growth of beneficial bacteria could potentially enhance the SCFA production and thus improve energy efficiency, enabling better growth rates and feed conversion ratio.

### 4.2 Immunoregulation

In poultry farming, maintaining robust immune function is essential to prevent disease outbreaks, reduce mortality and optimize production efficiency. The bioactive compounds of AP are suggested to modulate immune response, making it a valuable natural alternative to synthetic antibiotics for enhancing poultry health.

The immunomodulatory effect of AP has been well-documented in research focused on its ability to enhance both innate and adaptive immunity. AP was shown to significantly enhance humoral immunity in laying hens, demonstrated by an increase in anti-Newcastle Disease, infectious Bursal Disease (Mathivanan and Kalaiarasi, 2007), and avian influenza antibody titers (Darmansyah et al., 2024). Same effect against Newcastle Disease virus was observed when tested in-ovo (Arify et al., 2018a; Baskaran et al., 2020; Nagajothi et al., 2020).

The AP supplementation was also related to improved serum parameters such as higher lymphocyte conversion rate and serum lysozyme levels in ducks (Liu et al., 2023), directly indicating stronger immune activity, as lymphocytes are critical in adaptive immunity, while lysozyme acts as an antimicrobial enzyme that reduces pathogen load. In the same study, the enlargement of key immune organs such as the thymus and the bursa of Fabricius was observed. These organs play critical roles in the development and function of the avian immune system, particularly in young birds whose immune systems are still maturing. Feed fortification with AP extracts also has a positive impact on blood immune parameters such as the white blood cells and lymphocyte count (Arify et al., 2018b), which further supports the adaptive immune system by ensuring that the birds can mount a quick and strong response to reinfections.

Some studies have indicated that supplementation of broiler feed with AP can improve immune cell profiles in poultry, particularly under conditions of bacterial and parasitic infections. For instance, Hidanah et al. (2023a) reported that AP supplementation helped restore immune cell counts such as leukocytes and heterophils, which are typically elevated in response to infection, while reducing basophils in *Salmonella*-infected broilers. By restoring these cell counts, AP supplementation helps normalize immune function, aiding in recovery and protection against further damage. In addition, AP has been shown to mitigate the negative impacts of *E. coli* infections by improving performance parameters of laying hens like feed consumption, feed conversion, and egg production (Hidanah et al., 2020). Jahja et al. (2023) found that AP supplementation in broilers had the anticoccidial effect, which was manifested by a significant reduction of the number of *Eimera tenella* oocysts/g of feces after infection with live coccidia vaccine. Moreover, AP extracts can reduce the motility of *Ascaridia galli*, a pathogenic parasite of poultry, collected from the intestinal lumen of free-range chickens, which indicates its antiparasitic effect (Tiuria et al., 2024).

### 4.3 Hepatoprotective effects

The liver is a vital organ, playing a central role as the indicator of metabolism level and pathological processes in birds (Skovorodin et al., 2019). In commercial poultry production, the liver often faces high metabolic demands and is susceptible to damage from oxidative stress, toxins, and pathogens. Therefore, maintaining the liver in an excellent condition is essential to ensure overall health and productivity in poultry (Zaefarian et al., 2019).

*A. paniculata* is known for its hepatoprotective effects, which have also been confirmed in birds, especially in the case of aflatoxin poisoning. Aflatoxins can affect not only the liver and digestive tract, but also immune and nervous systems (Chałaśkiewicz et al., 2025). Aneesh et al. (2020) verified that the addition of AP extracts to feed eliminated the macromolecular and oxidative damage caused by aflatoxin in the liver and kidney of broilers, such as cytoplasmic vacuolation, Kupffer cell hyperplasia, focal accumulation of mononuclear cells and acinar pattern of parenchyma. Similarly, Fayed et al. (2023) have observed the enhancement in liver weights and color in broiler chickens with afla- and ochratoxicosis supplemented with a herbal mixture containing AP compared to the mycotoxinated group. Also, the disappearance of degenerative changes in liver tissue (such as loss of regular hepatic organization with hydropic disintegration, vacuolations, and apoptotic hepatocytes) was observed. This study also showed that supplemented birds had improved liver function, which was manifested by the lowered blood plasma activity of known markers of liver damage—alanine aminotransferase (ALT) and aspartate aminotransferase (AST). Lowered alkaline phosphatase (ALP), ALT, and AST activity in blood were also observed in healthy chickens supplemented with AP (Malahubban and Aziz, 2018; Mathivanan and Kalaiarasi, 2007).

Moreover, Fayed et al. (2023) observed a reduction in caspase-3 (enzyme involved in apoptosis, indicating cellular damage or death) and TNF-α (pro-inflammatory cytokine) expression caused by mycotoxin-induced oxidative effect in broilers supplemented with AP. Addition of AP in the diet of mycotoxin affected broilers also increased the activity of hepatic antioxidant defense compounds such as glutathione reductase and glutathione peroxidase (Al-Zuhariy and Hassan, 2017) and modified the concentrations of hepatic oxidative stress biomarkers like malondialdehyde or reduced glutathione (Fayed et al., 2024). It is worth noting that increased antioxidant enzyme activity does not indicate liver damage, but rather enhanced oxidative defense, and therefore does not contradict the lowered ALT and ALP activity observed in healthy or supplemented birds in other studies. These findings are consistent with the broader strategies discussed in Kępka-Borkowska et al. (2025), which emphasizes the vital role of integrative mitigation approaches, including natural antioxidants and biological agents, in countering aflatoxin-induced damage and supporting liver health.

### 4.4 Regulation of lipid metabolism

The avian liver is a central organ regulating and maintaining the metabolism of lipids, playing critical roles in energy storage, synthesis and transformation of fats. Liver is also the main site of lipogenesis in avian species, where 90% of fatty acids are synthesized de novo (Emami et al., 2021). This organ's efficient functioning is fundamental to maintaining energy and metabolic balance. Some factors like heat stress (Lu et al., 2019) or hormonal imbalance caused by chronic stress (Duan et al., 2014) have been found to induce lipid synthesis, which is manifested by the high cholesterol and triglyceride levels in the liver, muscles and serum of broilers. Numerous studies indicate that AP supplementation in poultry leads to significant reductions in serum cholesterol and triglyceride levels (Arify et al., 2018b; Malahubban and Aziz, 2018; Mangisah et al., 2024; Mathivanan and Kalaiarasi, 2007; Paramasivam, 2024), potentially due to the presence of saponins and fiber that bind to serum lipids in the digestive system, especially cholesterol, thereby easing their excretion from circulation, and flavonoids which can reduce the activity of cholesterol synthesis in the liver. Maintaining balanced cholesterol and triglyceride levels minimizes the risk of metabolic disorders, reduces morbidity and potentially improves the overall welfare of poultry flocks.

### 4.5 Growth parameters and production traits

The positive effects of AP supplementation result in improved growth parameters and production traits either directly or indirectly. Studies indicate that birds fed diets enriched with AP show higher average daily gain (ADG) and higher final body weight (BW) (Al-Zuhariy and Hassan, 2017; Jahja et al., 2023; Liu et al., 2023; Paramasivam, 2024). Some researchers have also observed increased average feed intake (AFI) (Fayed et al., 2023; Paramasivam, 2024) in AP supplemented animals. It has to be pointed out that enhanced digestion and metabolism can lead to more consistent and predictable feed intake. AFI is strongly related to feed conversion ratio (FCR) that reflects the amount of feed consumed per unit of weight gained by the animal (Lakamp et al., 2022). The AP supplementation studies reported decreased FCR (Arify et al., 2018b; Fayed et al., 2023; Hidanah et al., 2020; Liu et al., 2023; Yulianti et al., 2015), which indicates higher feed utilization efficiency. Enhanced gut health, improved microbiota composition, increased nutrient digestibility and fortified immune function as an effect of AP supplementation result in the improvement of feed utilization efficiency and BW gain and final BW. Arify et al. (2019) calculated that by improving these parameters, the cost effectiveness of the broiler chicken fed with various levels of AP showed increased net profit per kg of live weight.

*A. paniculata* could also find applications in reducing stress caused by high cage density. Stress can alter the balance of intestinal bacteria, which is manifested by an increase in coliform counts in feces. Supplementation of AP causes the reduction of the number of intestinal coliform at high cage density (5 birds/m^2^) in ducks while maintaining daily weight gain comparable to control group (Mangisah et al., 2024).

Furthermore, AP supplementation had a positive impact on carcass quality parameters like carcass percentage, cooking loss, meat color, water-holding capacity and texture (Hasanain et al., 2024). A higher percentage of breast muscle with lower percentage of abdominal fat was also observed in ducks, cockerels and broilers (Gunawijaya et al., 2021; Liu et al., 2023; Watanasit et al., 2005). These improvements in carcass quality parameters not only enhance the sensory and nutritional attributes of the meat but also provide economic advantages across the supply chain.

Feed enrichment with AP also positively affected daily egg production in ducks (Yulianti et al., 2015) and laying hens (Hidanah et al., 2020). AP feed enrichment contributes to an increase in daily egg production, which is essential for improving overall productivity and profitability in poultry production. In addition to increasing egg production, AP supplementation has shown positive effects on egg quality, an important factor for both consumer satisfaction and economic value. Hidanah et al. (2023b) confirmed that AP not only increased egg size, but also enhanced the quality of the eggs, increasing both the yolk and the albumen. This alignment of consumer preferences with production efficiency ensures better market acceptance and profitability.

**Fig. 3:**
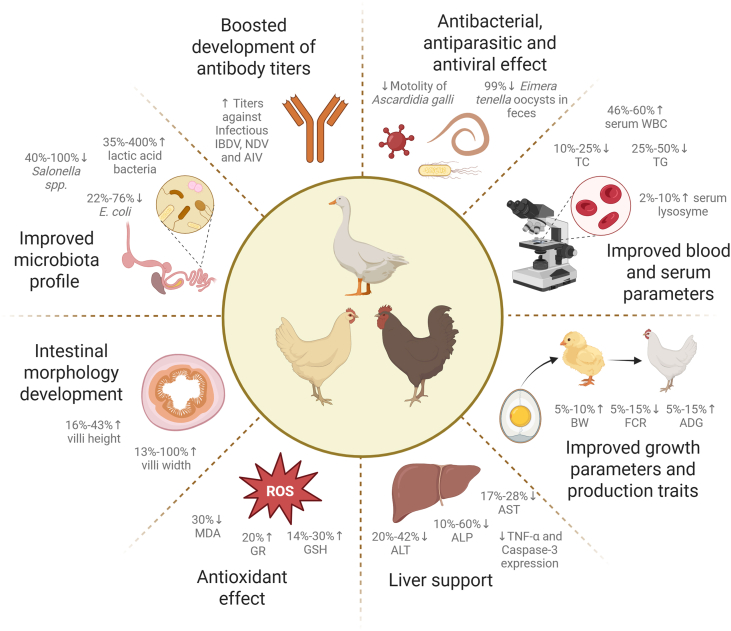
The summary of effects of *Andrographis paniculata* (AP) supplementation in poultry. AIV = avian influenza virus; NDV = newcastle disease virus; IBDV = infectious bursal disease virus; ALP = alkaline phosphatase; ALT = alanine transaminase; AST = aspartate aminotransferase; ADG = average daily gain; FCR = feed conversion ratio; BW = final body weight; TC = total cholesterol; TG = total triglycerides; WBC = white blood cells count; GR = glutathione reductase; GSH = glutathione; MDA = malondialdehyde.

In summary, AP offers significant benefits in poultry. As a natural feed additive, AP supplementation has the potential to improve metabolic efficiency, support growth performance, and promote a healthier physiological profile, making it a promising option in sustainable poultry nutrition and health management. The bunch of evidence has also confirmed its usefulness in the replacement of antibiotic growth promoters. The wide spectrum studies summarizing the effects of AP supplementation in poultry have been given in Table 1. Additionally, the main observed effects of AP supplementation in poultry have also been visualized in Fig. 3.

**Table 1:** Summary of the studies investigating the supplementation of *Andrographis paniculata* (AP) in poultry.

Animals	Health status	Doses	Duration, d	Outcomes	References
Laying hens	Vaccinated against AIV and NDV	5000 mg/L of leaf extract in drinking water (ad libitum)	24	Boosted the development of anti-AI and -ND antibody levels	Darmansyah et al., (2024)
	Infected with *Escherichia. coli*	300 mg/bird of AP extract	28	Eliminated the negative effects of infection. Restored post-infectious egg production and decreased FCR	Hidanah et al., (2020)
	Infected with *E. coli*	200 mg/bird of AP extract	21	Improved the egg yolk index and HU value	Hidanah et al., 2023a, Hidanah et al., 2023b
Broilers	Healthy	15 mg/kg feed of AP extract	28	Improved BW and FCR, improved microbiota profile increased *Lactobacillus* spp. and *Bacillus* spp. and decreased *E*. *coli* and *Salmonella* spp. count	Jahja et al., (2023)
	Infected with *Eimeria* spp.	15 mg/kg feed of AP extract	28	Reduced number of *Eimeria* fecal oocysts	Jahja et al., (2023)
	Aflatoxicosis	2000 mg/kg feed of AP powder	21	Diminished aflatoxicosis histological effects damage in kidney and liver – reduced number of cytoplasmatic vacuolations, Kupffer cell hyperplasia, focal accumulation of mononuclear cells and acinar pattern of parenchyma and increased lipid peroxidation levels	Aneesh et al., (2020)
	Healthy	2000 mg/kg feed of AP powder	42	Increased white blood cells and lymphocyte count in blood, reduced TC and TG levels in serum	Arify et al., (2018b)
	Infected with *Salmonella Pullorum*	50 mg/bird of AP extract	14	Restored number of leukocytes, heterophils and reduced basophils in blood	Hidanah et al., 2023a, Hidanah et al., 2023b
	Healthy	7500 mg/kg feed of AP extract	–	Improved jejunal microbiota profile by increasing the number of lactic acid bacteria and decreasing the number of *E*. *coli* and *Salmonella*	Hasanain et al., (2023)
	Healthy	8000 mg/kg body weight of AP leaf	21	Decreased ALP and ALT activity in blood, reduced TC and TG levels in serum	Malahubban and Aziz (2018)
	Healthy	2000 mg/kg feed of AP powder	42	Improved ADG and AFI, reduced serum TC level	Paramasivam (2024)
	Healthy	2000 mg/kg feed of AP powder	42	Improved ADG and FCR, increased net profit due to cost effectiveness	Arify et al., (2019)
	Afla- and ochratoxicosis	500 mg/kg feed of herb-all liver (mainly *A. paniculata*, *T. cordifolia*, and hickory nuts)	35	Improved liver–lower activity levels of ALT and AST, lower TC and HDL levels in blood; enhanced liver weight and color, improved liver architecture, reduced Caspase-3 and TNF-α expression, increased malondialdehyde level and decreased reduced glutathione activity, improved ADG and FCR	Fayed et al., (2023)
	Aflatoxicosis	2000 mg/kg feed of AP powder	45	Improved ADG and FCR, increased level of hepatic and spleen antioxidant defense compounds – glutathione reductase and glutathione peroxidase	Al-Zuhariy and Hassan (2017)
	Healthy	2000 mg/kg feed of AP leaf powder	42	Decreased TC level and AST and ALT activity in serum, increased antibody titers against NDV and IBDV	Mathivanan and Kalaiarasi (2007)
	Healthy	2500 mg/kg feed of nano liquid extract with AP	35	Improved body weight, FCR and carcass quality parameters – carcass percentage, cooking loss, meat color, water-holding capacity and texture	Hasanain et al., (2024)
	Infected with *Ascaridia Galli*	0,5 mg/worm of AP extract	–	Reduced the motility of *A. Galli* collected from the intestinal lumen	Tiuria et al., (2024)
	Healthy	2000 mg/kg feed of AP powder	42	Decreased the percentage of abdominal fat	Watanasit et al., (2005)
	Healthy	2000 mg/L of AP leaf extract in drinking water (ad libitum)	49	Decreased the percentage of abdominal fat, increased the percentage of breast muscle	Gunawijaya et al., (2021)
Endemic chicken breed In ovo	Healthy	3000 mg per bird of AP powder	60	Decreased crypt depth and increased villi height in the duodenum in the progeny	Sujatha et al., (2015)
	–	2 mg/egg of ethanolic AP extract	–	Antivirus activity against NDV	Arify et al., (2018a)
	–	0.120 mg/egg of methanolic AP leaf extract	–	Antivirus activity against NDV	Baskaran et al., (2020)
	–	0.025 μg/egg of aqueous AP leaf extract (total inhibition)	–	Antivirus activity against NDV	Nagajothi et al., (2020)
		0.025 μg/egg of isolated AG (total inhibition)			
		0.025 μg/egg of ethanolic AP leaf extract (partial inhibition)			
Ducks	Healthy	10 000 mg/kg feed of fermented AP	70	Increased villi height, width, intestinal thickness and surface area, improved serum parameters – lymphocyte conversion rate and serum lysozyme, enlarged immune organs – thymus, bursa of Fabricus, improved cecal microflora profile – decreased the relative abundance of harmful bacteria (*Succinivibrio*, *Succinatimonas*, *Sphaerochaeta*, and *Mucispirillum*) and increased the abundance of beneficial bacteria (Rikenellaceae, *Methanocorpusculum*, *Fournierella*, Ruminococcaceae); improved ADG and FCR and carcass quality parameters – increased the dressed percentage and the percentage of breast muscle	Liu et al., (2023)
	Healthy	2000 mg/kg feed of AP leaf powder	–	Improved FCR and egg production	Yulianti et al., (2015)
	High cage density	5000 mg/kg feed of AP leaf powder	28	Improved blood parameters – decreased TC and LDL levels, improved BW; reduced coliforms caused by high cage capacity stress	Mangisah et al., (2024)

AG = andrographolide; AIV = avian influenza virus; NDV = newcastle disease virus; IBDV = infectious bursal disease virus; ALP = alkaline phosphatase; ALT = alanine transaminase; AST = aspartate aminotransferase; ADG = average daily gain; FCR = feed conversion ratio; AFI = average feed intake; BW = body weight; TC = total cholesterol; TG = total triglycerides.

## 5 Effects of AP supplementation in pigs

Pigs play a significant role in global meat production, making it imperative to investigate innovative strategies to improve their growth, feed efficiency, and overall health (Kim et al., 2023). While research on AP applications in poultry has expanded considerably in recent years demonstrating multifarious benefits, the exploration of its effect in pigs remains relatively limited. This disparity highlights a critical knowledge gap in understanding the potential of AP supplementation in swine nutrition and health management. Most research is only focused on growth performance, without taking into account the broad spectrum of AP action in the digestive or immune system.

### 5.1 Sows

The potential of supplementing AP in sows represents an important area of investigation. Sows play a pivotal role in the productivity and health of a swine operation, as their nutritional and health status directly influence reproductive performance, litter outcomes and the early development of piglets. Most research on the application of AP in sows has focused primarily on the farrowing and lactation phases, the most critical periods.

Sows fed AP fortified feed during lactation exhibited significant improvements in the number of piglets weaned and their individual weights, as well as increased overall litter weight gain (Tummaruk and Limtrajitt, 2010). Additionally, it enhanced the AFI of sows during the lactation period, supporting better maternal health and nutrient transfer to the piglets. Similar results were obtained in preliminary research by Tummaruk et al. (2009), where sows supplemented with AP had an improved number of piglets at weaning, litter weight gain and pre-weaning mortality rate. Tummaruk et al. (2011) also showed that AP could be supplemented in feed of the pregnant gilts and sows without any decrease in the progesterone level during early pregnancy. Furthermore, sows receiving AP supplementation experienced reduced loss of longissimus dorsi muscle depth during lactation, indicating better preservation of body condition and reduced catabolic stress during this demanding period (Tummaruk et al., 2024). AP-enriched feed also elevated immunoglobulin G (IgG) levels in sow colostrum post-farrowing, which is crucial for bolstering piglet immunity. Additionally, piglets born to AP-supplemented sows showed increased colostrum intake, which positively influences their survival rate and early growth. Colostrum is a critical factor for neonatal immunity and its enhanced composition promotes better piglet energy supply, gut and passive immunity development (Farmer et al., 2006).

The application of AP has also been explored under heat stress conditions, which is known to negatively affect sow reproductive performance and piglet survival. AP supplementation in primiparous sows reduced the number of stillborn piglets and increased the number of piglets surviving 24 h post birth, as well as the number of piglets weaned successfully (Papatsiros et al., 2022). Moreover, application of AP under heat stress was associated with improved post-weaning reproductive parameters. Sows treated with AP exhibited a shortened weaning-to-estrus interval, a key indicator of reproductive efficiency, alongside better body condition scores and thicker back fat a reflection of improved nutritional recovery and energy reserves. Supplemented animals also displayed lower levels of lipid peroxidation (measured by thiobarbituric acid reactive substances, TBARS) and protein oxidation (indicated by carbonyl levels, CARB). These findings suggest that AP can help mitigate the detrimental effects of heat stress, potentially by reducing oxidative stress, improvement of sow resilience as well as maternal performance.

These beneficial reproductive outcomes can be attributed to the bioactive effects of AP on maternal physiology. AG exerts anti-inflammatory action through inhibition of NF-κB signaling and downregulation of cytokines such as TNF-α and IL-6, which are known to impair ovarian function and delay estrus return (Orisaka et al., 2023). Additionally, AP enhances antioxidant capacity of reproductive tissues, which are vulnerable to oxidative damage, especially under high metabolic demand (Yang et al., 2023). Furthermore, elevated IgG levels in colostrum suggest improved maternal immune status and nutrient partitioning, facilitating enhanced passive immunity and piglet viability. Improved feed intake and reduced catabolic stress during lactation likely support better body condition and metabolic recovery, which are crucial for timely return to estrus and fertility post-weaning. These multifactorial effects collectively contribute to improved reproductive performance and sow resilience.

### 5.2 Piglets

Research on AP supplementation in pigs largely focuses on piglets. Intensively raised piglets face numerous challenges during early life, including weaning stress, susceptibility to diseases, and underdeveloped digestive and immune systems (Prunier et al., 2010). These stressors can significantly impact growth performance, feed efficiency and overall health. The application of AP in piglets represents a promising strategy to address critical challenges faced during early piglet development, especially during weaning period.

He et al. (2024) demonstrated that AG treatment can be effective against porcine epidemic diarrhea virus (PEDV). Supplementation of this bioactive compound in infected piglets reduced clinical symptoms of infection such as diarrhea, increased body temperature and maintained mean body weight throughout the experiment with no significant difference from the control group. It also ameliorated intestinal damage (mucosal injury, hemorrhage and edema, necrosis and vacuolation) and increased the survival rate of infected animals. The viral loads of the jejunum, ileum, and anal swabs in animals treated with AP were significantly lower. Additionally, the expression of the genes for pro-inflammatory cytokines (IL-6, TNF-α, and IL-1β) were decreased. These findings underline AP's potential as a natural therapeutic agent against PEDV (Jung et al., 2020).

In contrast to studies on sows, research in piglets indicate the potential use of AP as a component in multi-herbal preparations, which positively influences the growth performance. Herbal mixture containing AP (Natu B4) increased the piglets' weight after 40 d of feeding compared to the control group, with a simultaneous improvement in FCR (Atkinson et al., 2020). Similar positive effects of AP supplementation on growth performance have been reported in earlier studies. For instance, Lipiński et al. (2011) observed that weaned piglets fed a diet enriched with a herbal blend including AP exhibited higher ADG and improved FCR compared to unsupplemented individuals. Likewise, Porntrakulpipat et al. (2010) reported that piglets from sows supplemented with a herbal mixer (Herbatob-Mix) containing AP achieved increased ADG after 21 d of suckling period. By improving ADG and reducing FCR, these blends not only promote growth and general health status of piglets but also offer economic advantages to producers, particularly in systems seeking alternatives to antibiotic growth promoters.

*A. paniculata* has shown considerable promise in aiding piglets to combat bacterial infections, particularly those caused by *E. coli*, a major pathogen in swine production (Barros et al., 2023). Herbal mixture containing AP (MuPlus) reduced the number of hemolytic *E. coli* colonies in feces of infected weaned piglets and alleviated the symptoms of infection by relieving diarrhea and increasing the villi height in all parts of the small intestine (Laxaphakdy et al., 2023). Also, Siriwathananukul et al. (2010) highlighted AP's therapeutic potential in addressing *E. coli-*induced diarrhea in suckling pigs supplemented with 0.5 g AP leaves 2 doses/d combined with *Psidium guajava* leaves. The antidiarrheal effect of AP may be attributed to multiple mechanisms reported in another species. AG has broad-spectrum antibacterial effects, particularly against enterotoxigenic *E. coli* by influencing the quorum sensing system or inhibiting bacterial adhesion and integrity, which can help reduce pathogen load in the gut (Zhang et al., 2019). Also, AP may reduce the mucosal inflammation though downregulation of cytokines like TNF-α and IL-6 (Yin et al., 2022). Furthermore, AP supports intestinal integrity by promoting villus regeneration (Laxaphakdy et al., 2023), modulating gut microbiota (Jahja et al., 2023), and enhancing local antioxidant defenses (Shi et al., 2020), all of which contribute to improved barrier function and reduced fluid loss. By reducing pathogen burden, alleviating inflammatory damage, and promoting intestinal repair, AP can improve not only the health status of infected piglets but also their overall productivity.

### 5.3 Grower pigs

The least studied group in terms of the positive impact of AP supplementation are grower pigs. These animals face a unique set of challenges during their development, including transitioning to solid feed, rapid growth demands and exposure to environmental and nutritional stressors. As these animals enter the post-weaning phase and transition into market weight, their nutritional and health requirements evolve, and their performance becomes increasingly critical for production efficiency and profitability.

The results observed in grower pigs supplemented with AP are consistent with findings from studies on sows and piglets, demonstrating improvements in growth performance. Gyani et al. (2016) reported that grower pigs receiving a herbal product containing AP exhibited several key benefits, including significantly higher final body weight and ADG from the first to the sixth fortnight of the experiment. In addition to these core growth metrics, physical development parameters such as body length, chest girth, and height at the withers were also improved, indicating enhanced structural growth and overall robustness. Furthermore, these improvements were accompanied by a reduction in FCR.

Beyond growth performance, Lipiński et al. (2011) provided additional insights into the nutritional benefits of AP supplementation. Their study found that the herbal blend containing AP enhanced the digestibility of total protein and crude fat in young barrows. Enhanced protein and fat digestibility are particularly valuable during the grower phase, as these nutrients are critical for supporting rapid growth and muscle development while minimizing feed wastage. These findings suggest that AP may improve digestive efficiency. The observed benefits align with those seen in piglets and sows, highlighting the consistency of AP's positive effects across various physiological stages of swine production.

Several studies evaluating AP in piglets and grower pigs utilized it not as a standalone supplement but as part of multi-herbal blends, suggesting possible synergistic interactions with other phytogenic compounds. For instance, Gyani et al. (2016) and Lipiński et al. (2011) observed enhanced growth performance and nutrient digestibility in grower pigs when AP was combined with other herbs such as *Allium sativum*, *Azadirachta indica*, and *Terminalia chebula*. These synergies likely arise from complementary actions on gut health, immune modulation, and metabolic pathways. Bioactives from other herbs, such as flavonoids, tannins, and saponins, can enhance AP's effects by broadening antimicrobial spectra, potentiating antioxidant activity, or supporting gut barrier integrity. Additionally, in piglets, formulations like MuPlus and Natu B4, containing AP, have shown promising results in piglets and grower pigs, including improved ADG, FCR and gut morphology. While these formulations appear effective, current studies do not provide direct comparisons with single-herb AP treatments, making it difficult to determine the superiority of the mixtures. However, the use of AP in combination with other phytogenic feed additives may yield additive or synergistic effects, particularly when targeting multiple physiological pathways such as antimicrobial activity, antioxidant defense, and mucosal immunity. Further research is warranted to explore these interactions, define optimal combinations, and validate synergistic benefits under controlled conditions.

### 5.4 In vitro and in silico approaches

Except in vivo studies including different production stages, a deeper understanding of AP's mechanisms of action can be gained through in vitro or in silico studies. In vitro methodologies provide controlled conditions to investigate and predict AP's properties at cellular level, while in silico modeling offers predictive insight into its biochemical interactions and pharmacokinetics. These approaches complement in vivo studies, creating a basis for possible future research.

All studies of that type concern the action of bioactive substances contained in AP on viruses that severely impact swine health and production. In vitro experiments carried out by He et al. (2024) explored the action of AG in inhibiting PEDV replication. The proposed mechanism involves the suppression of PEDV-induced activation of the JAK2-STAT3 signaling pathway, a crucial pathway that facilitates viral propagation and promotes apoptosis in host cells. Su et al. (2021) demonstrated that AG exhibit robust activity against porcine reproductive and respiratory syndrome virus (PRRSV) replication in Marc-145 cells and primary porcine alveolar macrophages. AG was found to suppress PRRSV replication effectively, which was linked to its potent inhibition of the NF-κB signaling pathway, and to mitigate the oxidative damage associated with PRRSV infections.

However, it is important to note that the effective concentrations reported in vitro, such as 50 μM of AG (He et al., 2024), are not readily achievable through conventional dietary supplementation in swine. When translated into practical feeding levels, such concentrations would require unrealistically high doses, well beyond the 10 to 100 mg/kg feed range commonly tested in vivo. Furthermore, physiological factors such as absorption, metabolism, and tissue distribution limit the systemic availability of AG following oral administration. This highlights a key translational limitation, as the antiviral effects observed in vitro may not directly correspond to outcomes in whole-animal systems without optimized delivery strategies or pharmacokinetic enhancements.

In silico studies have also emerged as powerful tools for exploring the pharmacological potential of bioactive compounds found in AP. The computer modeling showed that AGP and NAG were found to effectively inhibit swine flu (H1N1) neuraminidase by binding to the active site of the protein (Sowmya and Sudharshan, 2023). Similar virtual screening and molecular docking simulations by Seniya et al. (2014) suggested that AG also has the potential to inhibit the neuraminidase activity of H1N1. AG was even found to be the most fitted ligand with the active side residues than the approved drug for swine flu. While these simulations suggest a strong pharmacological potential, their predictive nature requires cautious interpretation. In silico outcomes are based on idealized binding affinities and do not account for physiological barriers, compound bioavailability, or complex host–pathogen interactions that can influence real-world efficacy.

**Fig. 4:**
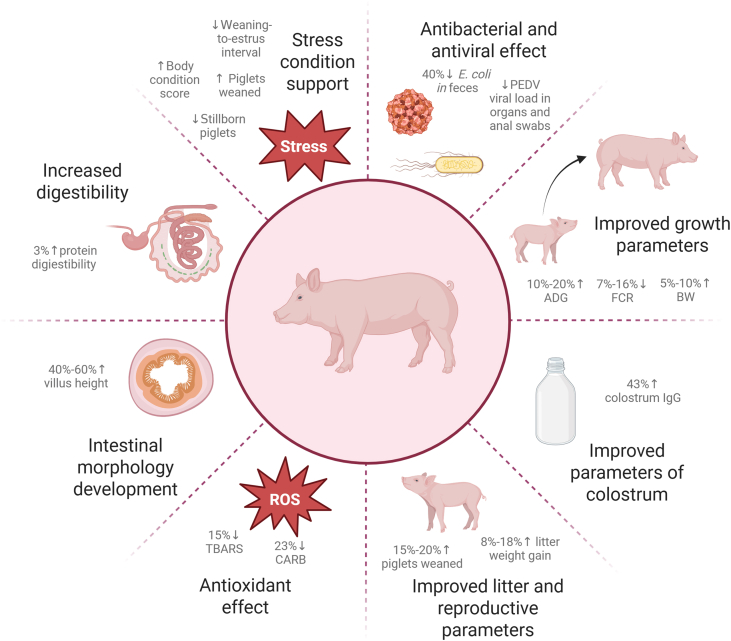
The summary of effects of AP supplementation in pigs. PEDV = porcine epidemic diarrhea virus; ADG = average daily gain; FCR = feed conversion ratio; BW = final body weight; IgG = immunoglobulin G; TBARS = thiobarbituric acid reactive substances; CARB = indicated by carbonyl levels.

In summary, AP offers a natural, multifunctional solution for addressing key challenges in pig production, including growth performance, disease management, and stress resilience. Its application supports the ongoing shift toward sustainable and health-focused practices in the swine industry, benefiting both producers and consumers. Continued research and innovation will further unlock its full potential, cementing its role as a vital tool in modern swine husbandry. The summary of the studies investigating supplementation of AP and their effects in pigs are shown in Table 2 and Fig. 4.

**Table 2:** Summary of the studies investigating the supplementation of *Andrographis paniculata* (AP) in pigs.

Animals	Health status	Doses	Duration, d	Outcomes	References
Sows	Healthy	1000 mg/kg feed of AP powder	26 to 38	Increased colostrum intake and colostrum IgG, feed intake, decreased longissimus depth loss	Tummaruk et al. (2024)
	Heat stress conditions	5000 mg/kg feed of plant mixer with AP	14	Improved litter parameters – decreased the number of stillborn piglets, increased the number of piglets that remained alive after 24 h and weaned; improved body condition score and backfat, decreased oxidative stress-induced damage – decreased TBARS and CARB levels	Papatsiros et al. (2022)
	Healthy	3000 mg/d of herbal mixer	35 to 49	Improved ADG of piglets	Porntrakulpipat et al. (2010)
	Healthy	1000 mg/kg feed of AP powder	28	Improved litter parameters – the number of piglets at weaning, piglet weaning weight and litter weight gain; improved ADG of sows	Tummaruk and Limtrajitt (2010)
	Healthy	1000 mg/kg feed of AP powder	28	Improved litter parameters – the number of piglets at weaning, litter weight gain and pre-weaning mortality rate	Tummaruk et al. (2009)
	Healthy	500 mg/kg feed of AP powder	During pregnancy	No influence on fecal progesterone during early pregnancy period	Tummaruk et al. (2011)
Piglets	PEDV-infected	10 mg/kg feed of AG	9	Reduced clinical symptoms of infection – diarrhea, increased body temperature, reduced body weight; ameliorated intestinal damage – jejunal intestinal mucosal injury, hemorrhage and edema, intestinal necrosis and vacuolation in the ileum; increased the survival rate, decreased the viral load in jejunum and ileum, decreased the mRNA levels of pro-inflammatory cytokines – IL-6, TNF- α and IL-1β	He et al. (2024)
	Infected with *Escherichia coli*	1000 mg/kg feed of herbal mixture with AP	24	Decreased number of *E. coli* in feces, increased duodenal, jejunal and ileal villi height, lower fecal scores (less diarrhea)	Laxaphakdy et al. (2023)
	Healthy	250 mg/kg feed of herbal mixture with AP	42	Improved body weight and FCR	Atkinson et al. (2020)
	Healthy	1000 mg/kg feed of herbal mixture with AP	30	Improved ADG and FCR	Lipiński et al. (2011)
	Infected with *E. coli*	1000 mg/d of AP leaves	Variable – until recovery	Treated diarrhea caused by *E. coli*	Siriwathananukul et al. (2010)
Grower pigs	Healthy	2000 mg/kg feed of herbal mixer with AP	90	Improved body weight, ADG, body length, chest grith, height at withers and FCR	Gyani et al. (2016)
Fatteners	Healthy	1000 mg/kg feed of herbal mixture with AP	30	Improved total protein and crude fat digestibility	Lipiński et al. (2011)
In vitro	–	50 μmol/L of AG	–	Inhibited PEDV	He et al. (2024)
	–	126.8 μmol/L of AG	–	Inhibited PEDV	Su et al. (2021)
In silico	–	AGP and NAG	–	Inhibited neuraminidase of H1N1	Sowmya and Sudharshan (2023)
	–	AG	–	Inhibited neuraminidase of H1N1	Seniya et al. (2014)

AG = andrographolide; AGP = andrograpanin; NAG = neoandrographolide; PEDV = porcine epidemic diarrhea virus; H1N1 = swine flu virus; IgG = immunoglobulin G; ADG = average daily gain; FCR = feed conversion ratio.

## 6 Comparison and dose implications of AP supplementation

While direct comparative studies on the metabolism of AP between pigs and poultry are currently lacking, available evidence allows us to speculate on possible interspecies differences based on known physiological and pharmacokinetic characteristics. Pigs, as monogastric mammals, have a longer gastrointestinal transit time and more developed hepatic metabolism, which may lead to increased systemic absorption and biotransformation of AP compounds such as AG. In contrast, poultry exhibit a shorter digestive tract and faster intestinal passage, potentially limiting systemic exposure and favoring localized effects within the gut environment.

These physiological distinctions may help explain the differences in observed outcomes across species. In swine, AP has been linked to broader systemic effects, including improved reproductive performance, antioxidant status, and modulation of inflammatory markers in response to stress or mycotoxins. In poultry, the effects of AP are more frequently associated with gut-level outcomes such as improved intestinal morphology, reduced pathogen colonization, enhanced nutrient absorption, and lowered markers of liver damage. This suggests that while both species benefit from AP supplementation, the pathways and organs primarily influenced may differ. Additional research, particularly pharmacokinetic and tissue-distribution studies, is needed to clarify these mechanisms and refine species-specific dosing strategies.

Table 1, Table 2 clearly show that both poultry and swine studies demonstrate considerable heterogeneity in the dosing, formulation, and administration of AP, limiting the ability to establish precise, species-specific recommendations. However, despite these variations, some common patterns emerge that allow for cautious cross-species generalizations.

In poultry, AP extracts were effective at relatively low doses, typically in the range of 15 to 100 mg/kg feed, and were associated with improved growth performance, immune response, and microbial balance. In contrast, AP powder required significantly higher doses—commonly 2000 to 5000 mg/kg feed—to elicit comparable outcomes. Similar trends were observed in swine. For instance, supplementation with AP powder in sows at 1000 mg/kg feed consistently improved litter performance, colostrum quality, and sow recovery post-partum (Tummaruk et al., 2024). In piglets, AG—the primary bioactive compound—was effective at 10 mg/kg feed in mitigating virus infection (He et al., 2024), while herbal mixtures containing AP were generally used at 250 to 2000 mg/kg feed, resulting in improved gut morphology, reduced diarrhea incidence, and enhanced growth metrics.

These findings support the notion that formulation critically determines the effective dose. Extracts and purified compounds like AG tend to exhibit efficacy at lower inclusion rates due to higher concentrations of active constituents, whereas crude powders or herbal mixtures require higher dosages. Importantly, despite the wide dose range employed across studies (from 10 to 10 000 mg/kg), many reported benefits plateaued beyond moderate supplementation levels, suggesting non-linear dose–response relationships and possible saturation of biological effects.

Moreover, both species-specific and study-related factors (e.g., animal age, health status, baseline diet composition, and study duration) contribute to the observed variability. Differences in administration routes (per bird per d, per kg feed, or per L of water) and unclear reporting of treatment duration further complicate dose standardization. Based on the comparative review of available data, the following tentative dose recommendations can be proposed: extracts: 10 to 100 mg/kg feed and powders: 1000 to 2000 mg/kg feed. To enhance the real-world relevance of AP supplementation, it is useful to consider the production system context. In intensive farming systems, where animals face high stocking densities, elevated disease pressure, and greater heat stress, AP may serve as a natural alternative to synthetic antibiotics by improving immune resilience, modulating gut microbiota, and reducing oxidative stress. Its use may be particularly valuable in antibiotic-reduction programs or heat-stressed sow herds, where reproductive and metabolic outcomes are compromised. In free-range or organic systems, AP may be integrated as part of phytogenic feed strategies to support gut health and pathogen control without synthetic inputs. While administration through feed is most common in intensive settings, water-soluble extracts or encapsulated forms could be more suitable for extensive systems where precise feed intake is less predictable. Thus, tailoring the form and route of AP supplementation to the production model can enhance both effectiveness and practicality.

Future research should prioritize uniform dose units (e.g., mg/kg feed), quantify active ingredients such as AG, and include structured dose–response trials. Establishing biologically effective and economically feasible doses for different AP formulations will improve consistency across studies and support broader application in sustainable animal production.

## 7 Conclusions and perspectives

The supplementation of AP in monogastric farm animals has emerged as a promising approach to improve productivity, health and overall animal welfare. Across different species and production stages, AP has demonstrated a broad spectrum of benefits, including enhanced growth performance, digestive system support, improved immunity, and resilience against infections. Its hepatoprotective properties may also contribute to better animal health and improved quality of animal products. These effects are largely attributed to the bioactive compounds in AP, which possess well-documented antimicrobial, antiviral, antioxidant, and anti-inflammatory effects. The diverse benefits, combined with its potential to reduce dependence on synthetic antimicrobials and growth promoters, position AP as a key component in modern animal husbandry practices. However, its application in pigs under different environmental conditions is limited and warrants further investigation. Additionally, the standardization of AP formulations, optimal dosages, and long-term effects needs more attention to ensure consistent and safe use across various production systems.

## Credit Author Statement

**Elżbieta Redlarska:** Writing – review & editing, Writing – original draft, Visualization, Investigation, Conceptualization. **Małgorzata Ożgo:** Writing – review & editing. **Mariusz Pierzchała:** Writing – review & editing, Project administration, Funding acquisition. **Katarzyna Kępka-Borkowska:** Writing – review & editing. **Katarzyna Chałaśkiewicz:** Writing – review & editing. **Chandra Shekhar Pareek:** Writing – review & editing, Project administration, Funding acquisition. **Adam Lepczyński:** Writing – review & editing, Supervision, Project administration, Investigation, Funding acquisition, Conceptualization.

## Declaration of Competing Interest

We declare that we have no financial and personal relationships with other people or organizations that can inappropriately influence our work, and there is no professional or other personal interest of any nature or kind in any product, service and/or company that could be construed as influencing the content of this paper.
